# Intramolecular hydrophobic interactions are critical mediators of STAT5 dimerization

**DOI:** 10.1038/srep35454

**Published:** 2016-10-18

**Authors:** Dirk Fahrenkamp, Jinyu Li, Sabrina Ernst, Hildegard Schmitz-Van de Leur, Nicolas Chatain, Andrea Küster, Steffen Koschmieder, Bernhard Lüscher, Giulia Rossetti, Gerhard Müller-Newen

**Affiliations:** 1 Institute of Biochemistry and Molecular Biology, RWTH Aachen University, Aachen, Germany; 2 College of Chemistry, Fuzhou University, Fuzhou, China; 3 Computational Biomedicine, Institute for Advanced Simulation IAS-5 and Institute of Neuroscience and Medicine INM-9, Forschungszentrum Jülich, Jülich, Germany; 4 Department of Hematology, Oncology, Hemostaseology, and Stem Cell Transplantation, Faculty of Medicine, RWTH Aachen University, Aachen, Germany; 5 Jülich Supercomputing Centre (JSC), Forschungszentrum Jülich, Jülich, Germany

## Abstract

STAT5 is an essential transcription factor in hematopoiesis, which is activated through tyrosine phosphorylation in response to cytokine stimulation. Constitutive activation of STAT5 is a hallmark of myeloid and lymphoblastic leukemia. Using homology modeling and molecular dynamics simulations, a model of the STAT5 phosphotyrosine-SH2 domain interface was generated providing first structural information on the activated STAT5 dimer including a sequence, for which no structural information is available for any of the STAT proteins. We identified a novel intramolecular interaction mediated through F706, adjacent to the phosphotyrosine motif, and a unique hydrophobic interface on the surface of the SH2 domain. Analysis of corresponding STAT5 mutants revealed that this interaction is dispensable for Epo receptor-mediated phosphorylation of STAT5 but essential for dimer formation and subsequent nuclear accumulation. Moreover, the herein presented model clarifies molecular mechanisms of recently discovered leukemic STAT5 mutants and will help to guide future drug development.

STAT5, represented by the highly homologous isoforms STAT5A and STAT5B, is an essential transcription factor for the proliferation, survival and differentiation of myeloid cells^1^. Hematopoietic cytokines such as erythropoietin (Epo), thrombopoietin and GM-CSF activate Janus tyrosine kinases (JAKs) resulting in transient phosphorylation of STAT5 at a single conserved tyrosine residue (Y694 in STAT5A, Fig. 1a). Subsequently, STAT5 dimers are formed through reciprocal intermolecular phosphotyrosine-SH2 domain interactions. These dimers accumulate in the nucleus to induce target gene expression^2^,^3^. Persistently activated STAT5 has been observed in a variety of hematological disorders, including chronic (CML) and acute (AML) forms of myeloid leukemias^4^,^5^. Likewise, constitutive STAT5 signaling is associated with cKit^D816V^-positive systemic mastocytosis (SM)^6^,^7^ and several myeloproliferative neoplasms with mutated JAK2, MPL or calreticulin^8^. Importantly, STAT5 represents a vulnerable signaling node regulating tumor cell maintenance in the oncogenic networks of CML and AML cells, a feature known as non-oncogene addiction (NOA)^4^,^9^,^10^. Furthermore, the introduction of artificial gain-of-function mutants such as STAT5A^S710F^ into mice identified STAT5 as a putative oncogene capable of inducing tumor initiation in many hematopoietic cell types, independent of dysregulated upstream kinases^11^.

In addition to these functions in physiologic and malignant myelopoiesis, STAT5 represents a master regulator of lymphopoiesis, controlling the proliferation and differentiation of T and B cells through various cytokines including IL-2 and IL-7^12^,^13^. Accordingly, activating somatic mutations in the SH2 domain and the following C-terminal tail segment (CTS) of STAT5 (Fig. 1a) have been discovered in distinct types of T- and NK-cell lymphomas such as large granular lymphocytic leukemia (LGLL), acute T-cell leukemia (T-ALL) and T-cell prolymphocytic leukemia (T-PLL)^14^,^15^,^16^,^17^,^18^,^19^.

The addiction to STAT5-mediated signal transduction processes has been considered to be a potential Achilles heel in leukemic signaling networks, resulting in intense research aiming at the pharmacological inhibition of STAT5. Hence, prevention of STAT5 dimer formation is thought to interfere with tumor cell proliferation and survival^20^,^21^,^22^. However, advances in this field have been hampered by the lack of structural information on the active STAT5 dimer, which could have indicated molecular interactions amenable to small molecule interference. To understand the mechanisms that promote constitutive activation of STAT5 mutants and to generate a template that may guide structure-based drug discovery, we generated a molecular model of the STAT5 dimer interface through homology modeling and molecular dynamics simulations. The model was validated by functional analysis of point mutants revealing the importance of a unique hydrophobic interface on the SH2 domain of STAT5 for physiological as well as oncogenic activation of the transcription factor.

## Results

### Molecular dynamics simulation of the STAT5A dimer interface

**Figure 1:**
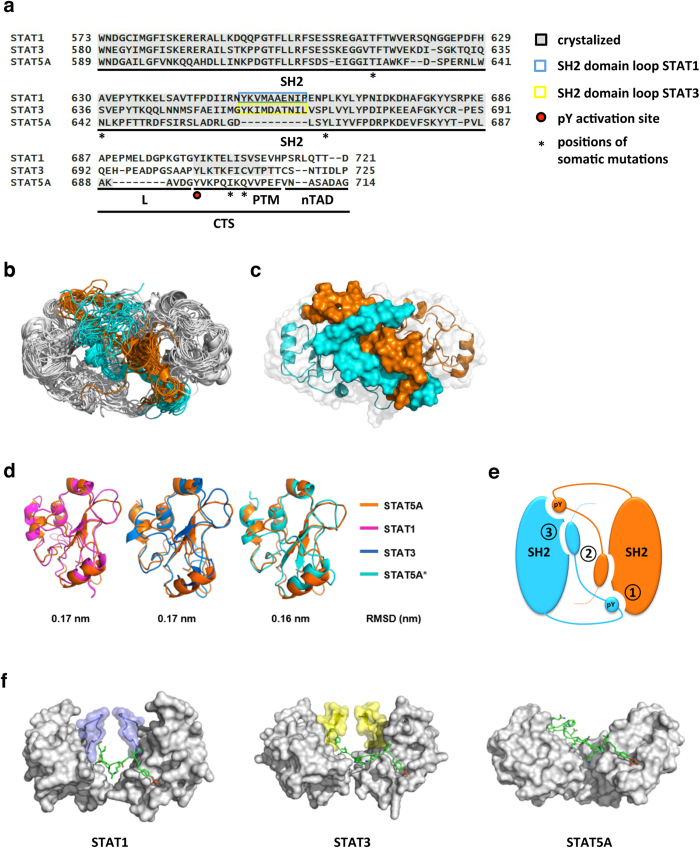
MD simulations of the STAT5A dimer interface. (**a**) Multiple sequence alignment of STAT1, STAT3 and STAT5A. SH2 domains and C-terminal tail segments (CTS) consisting of linker (L), phosphotyrosine motif (PTM) and the N-terminal part of the transactivation domain (nTAD) were aligned using the structure-guided alignment mode of T-coffee^56^. Crystalized regions are marked with grey background. The conserved phosphorylation site for activation of STAT proteins is highlighted by a red dot. SH2 domain loops contributing to dimerization of STAT1 and STAT3 are depicted in blue and yellow boxes, respectively. Positions of disease-related STAT5 mutations are indicated by asterisks. (**b**) Overlay of 20 frames (every 100 ns) of the MD simulations of the STAT5A dimer interface (top view). Grey: SH2 domains and L of monomer 1 (M1) and monomer 2 (M2). The PTM and nTAD (residues: 694–714) of monomers M1 and M2 are depicted in cyan and orange, respectively. (**c**) Most representative structure of the STAT5A dimer interface (top view) identified by cluster analysis over the equilibrated trajectories, ranging from 1500 ns to 2000 ns. SH2 domains and L are depicted as ribbon structures with transparent surface representation. PTM and nTAD are depicted as surface representation. Cyan: M1, orange: M2. (**d**) Superposition and root-mean-square deviation (RMSD) values between the most representative model of the activated STAT5A SH2 domain (M2, orange) and the SH2 domains of the crystal structures of activated STAT1 (pink)/STAT3 (blue) and inactivated (*) STAT5A (cyan) 23–25. (**e**) Cartoon of the STAT5A dimer interface. Interactions are established by three distinct interfaces involving intermolecular PTM-SH2 domain interactions (1), intermolecular PTM-PTM interactions (2) and intramolecular PTM-SH2 domain interactions (3). M1 and M2 are depicted cyan and orange, respectively. (**f**) Switch from inter- to intramolecular interactions in SH2 domain interfaces of STAT1, STAT3 and STAT5A (side view). SH2 domains are depicted as surface representation (grey) with SH2 domain loops colored in blue (STAT1) and yellow (STAT3) (compare Fig. 1a). The course of a single PTM of each dimer interface is shown in stick representation (green). Atoms color code: Green, carbon; red, oxygen; blue, nitrogen; orange, phosphorus; yellow, sulfur.

Oncogenic somatic STAT5 mutations are characterized by stimulation-independent activation and predominantly map to the SH2 domain (T628S, N642H, Y665F) of the transcription factor. However, constitutive STAT5 activation is additionally promoted by somatic mutations located in the sequence following phosphotyrosine 694 (I699L, Q701L), a region for which only limited or no structural information is available for any of the STAT dimers (Fig. 1a).

Therefore, the structural determinants of the STAT5A dimer interface were predicted by bioinformatics approaches and molecular dynamics (MD) simulations. The modeled system includes the SH2 domain (residues 589–687) and the C-terminal tail segment (CTS, res. 688–714), which consists of a short flexible linker (L, res. 688–693), the phosphotyrosine motif (PTM, res. 694–706) and the N-terminal part of the transactivation domain (nTAD, res. 707–714) (Fig. 1a). The initial model of the STAT5A active dimer interface was obtained by homology modeling. For the SH2 domain moiety the structure of the SH2 domain of inactive STAT5A^23^ as well as the available structures of the activated STAT1 and STAT3 dimers^24^,^25^ were used. Their SH2 domains share a very similar architecture with those of STAT5A with sequence identities of 30.8% and 34.2%, respectively (Supplementary Fig. 1a). For the CTS, which is not conserved across STAT proteins, we used templates other than STATs with sequence identities ranging from 26% to 37% (see Methods for more details).

Next, 2,000 ns-long MD simulations were performed (Fig. 1b) on the homology-based model of the active STAT5A dimer. Stability, sampling and convergence of the MD simulations were established by calculating the backbone root-mean-square deviation (RMSD) (Supplementary Fig. 1b). Hess analysis^26^ and RMSD both confirmed the adequate sampling of STAT5A conformations around the equilibrium position in the last 500 ns of the MD simulations (Supplementary Table 1). The most representative structure of the STAT5A dimer interface, covering more than half of the sampled conformations, was identified by cluster analysis (Fig. 1c). Superposition of our representative structure of the STAT5A SH2 domain revealed a high structural similarity with the crystalized SH2 domains of STAT1, STAT3 and inactivated STAT5A^23^,^24^,^25^ demonstrated by low backbone RMSD values (<0.2 nm) (Fig. 1d and Supplementary Fig. 1c). According to our model and in analogy to the crystal structures of activated STAT1 and STAT3 dimers, the dimerization of STAT5A is established by three distinct interfaces, involving reciprocal intermolecular PTM-SH2 domain interactions (1), intermolecular PTM-PTM interactions (2) and intramolecular PTM-SH2 domain interactions (3) (Fig. 1e). The first interface is characterized by the classical phosphotyrosine-SH2 domain interaction present in all activated STAT dimers, whereas the second and third interfaces are substantially different from those of STAT1 and STAT3. The latter indeed feature a flexible loop structure, which significantly contributes to the dimerization by providing a stabilizing framework for the percolating PTM (blue and yellow loops in Fig. 1f). Interestingly, the residues belonging to this loop-structure are not conserved in the SH2 domain of STAT5 proteins (blue and yellow boxes in Fig. 1a). Hence, the MD simulations suggest that the dimerization interface established by STAT5 proteins is different from those of STAT1 and STAT3.

### SH2 domain mediated STAT5 dimerization

**Figure 2:**
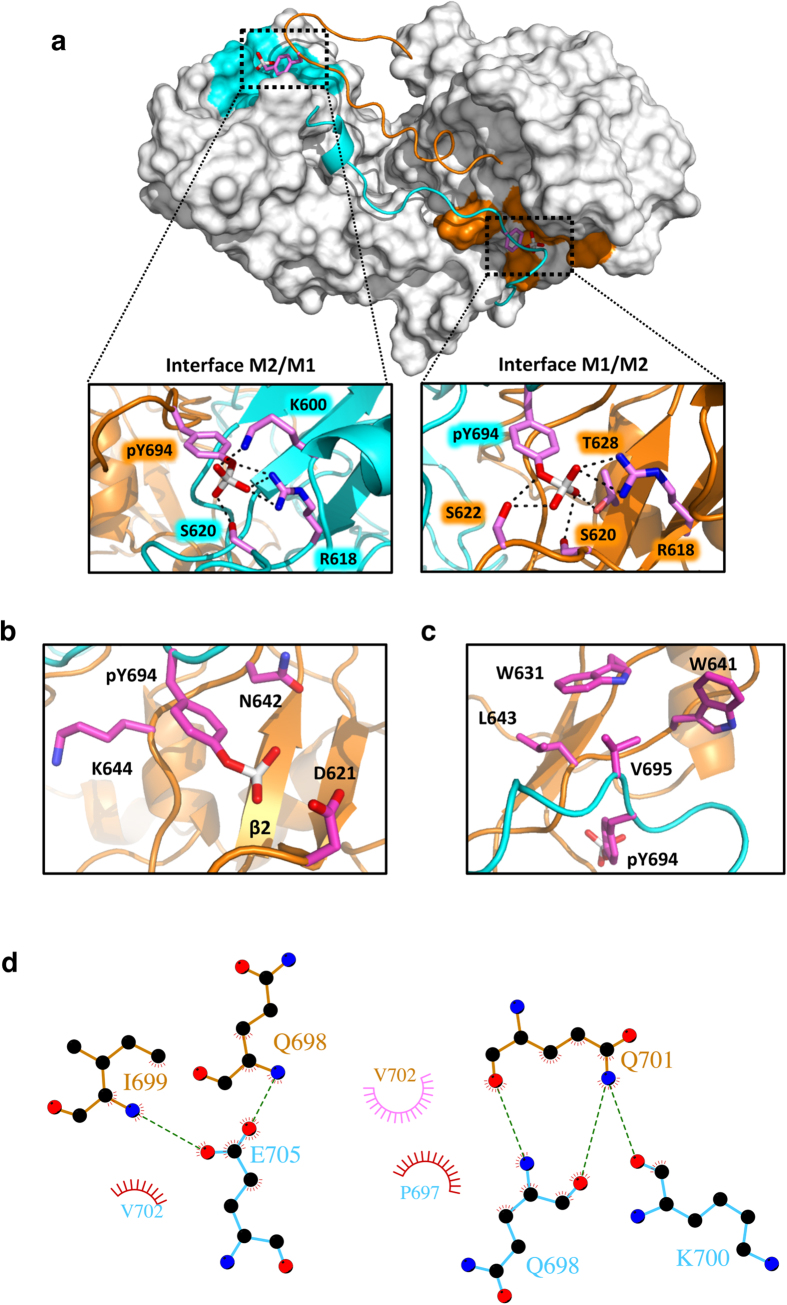
The phosphotyrosine-SH2 domain interface of STAT5A. (**a**) STAT5A dimer interface (top view). The SH2 domains are depicted in surface representation colored in grey, while the C-terminal part of the L, the PTM and the nTAD are shown as ribbon structure in cyan (M1) and orange (M2). The phosphotyrosine acceptor interfaces are highlighted in cyan (M1) and orange (M2). The side chain of pY694 is shown in stick representation. A magnification of the phosphotyrosine-SH2 domain interaction is shown for both interfaces. Selected residues are shown in stick representation. Side chains involved in pY694 coordination are depicted in magenta (carbon). Red, oxygen; blue, nitrogen; white, phosphorus. Residue labels are colored in cyan or orange according to the corresponding monomer. Hydrogen bonds are indicated by dashed lines (See also Supplementary Fig. 2 and Supplementary Table 2). (**b**) Magnification of the hydrophobic phosphotyrosine-SH2 domain interactions in M2. Residues involved in the coordination of phosphotyrosine 694 are shown in stick representation. N642 and K644 form direct hydrophobic contacts with the phenyl ring of phosphotyrosine. D621 localizes in close proximity to the phosphate group. The coloring scheme is the same as in (**a**). (See also Supplementary Fig. 2 and Supplementary Table 2). (**c**) Magnification of the V695-binding interface (pY+1) in the SH2 domain of M2. Residues forming hydrophobic contacts (W631, W641 and L643) with V695 are highlighted in stick representation. The coloring scheme is the same as in (**a**). (See also Supplementary Fig. 2). (**d**) Schematic, 2D representation of the STAT5A PTM-PTM interface generated by the LIGPLOT+ package^57^. Hydrophobic interactions (red, M1; pink, M2) and hydrogen bonds are indicated by spokes and dashed green lines, respectively. The residues forming hydrogen bonds are shown by ball-and-stick representations (cyan, M1; orange, M2).

In STAT5A, the phosphorylated tyrosine (pY694) in the CTS of monomer 1 (M1) interacts with the SH2 domain of the opposite monomer (M2) in a direction orthogonal to the strands of the central β-sheet (Fig. 2a). In a symmetric fashion, also pY694 in the CTS of M2 (pY694^M2^) interacts with the SH2 domain of M1 with the same orientation as pY694^M1^. Salt bridges formed by pY694 and the highly conserved and invariant R618 located in the βB strand of the opposite SH2 domain are key interactions in both intermolecular PTM-SH2 domain interfaces. Indeed, they are fully preserved during the MD simulations with occupancies of 100% (pY694^M1^) and 97% (pY694^M2^), respectively. Moreover, the coordination of pY694 in both monomers is supported by the formation of transient hydrogen bond interactions with K600, S620, S622 and T628 in the opposite monomer during the MD simulations (Fig. 2a and Supplementary Table 2). Notably, these residues are conserved in all STAT family members and have been described to be involved in the formation of hydrogen bonds in phosphotyrosine-SH2 domain interfaces of STAT1 and STAT3 (Supplementary Fig. 2 and Supplementary Table 3)^24^,^25^.

Our model further identified two novel residues involved in the recognition of pY694. These are the βD residues N642 (βD5) and K644 (βD7), which stabilize the aromatic ring of the phosphotyrosine by hydrophobic interactions (Fig. 2b and Supplementary Fig. 2). Finally, V695 (pY+1) packs into a hydrophobic pocket of the opposite monomer containing W631, W641 and L643, consistently to what has been observed in the structures of STAT1 and STAT3^24^,^25^ (Fig. 2c and Supplementary Fig. 2). We therefore conclude that our model of STAT5A correctly reproduces the conserved intermolecular PTM-SH2 domain contacts of known STAT dimer interfaces involving residues conserved across the family and at the same time reveals STAT5-specific interactions.

In addition to the PTM-SH2 domain interface, intermolecular interactions are observed between the C-terminal parts of the PTMs, allowing the recognition of each PTM by the opposite monomer over a length of 11 residues subsequent to phosphotyrosine. In contrast to the STAT1/STAT3 crystal structures, the PTM-PTM interface in our model is structurally disordered and constituted by non-conserved moieties. Although most of the interactions are transiently established due to the disordered nature of the two moieties, a distinct network of intermolecular hydrogen bonds was identified: this involves Q698^M1^ and K700^M1^ with Q701^M2^, and I699^M2^ and Q698^M2^ with E705^M1^ (Fig. 2d and Supplementary Table 4). Additionally, hydrophobic contacts between the side chains of P697^M1^ and V702^M2^, and between those of V702^M1^ and I699^M2^ were identified (Fig. 2d and Supplementary Table 5). Of note, residues in the pY+3 (P697), pY+5 (I699) and pY+7 (Q701) position ultimately define the shape of the PTM-PTM interface in STAT1 and STAT3 dimers.

### Intramolecular PTM-SH2 domain interactions: a hydrophobic interface in the SH2 STAT5 domain is critical for dimerization

**Figure 3:**
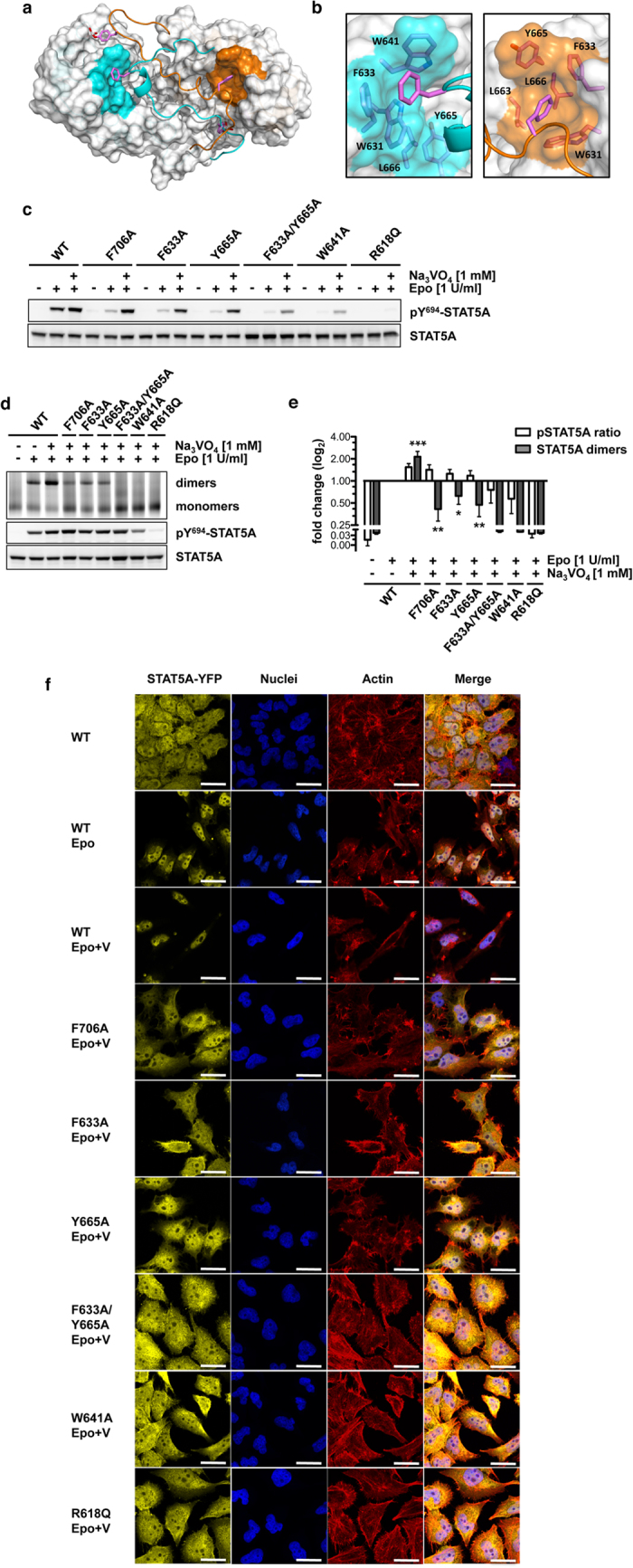
A hydrophobic interaction in the STAT5A SH2 domain is critical for dimerization. (**a**) STAT5A dimer interface (top view). The SH2 domains are depicted in surface representation (grey). The hydrophobic interfaces containing W631, F633, W641, L663, Y665 and L666 are highlighted in cyan (M1) and orange (M2). Both CTSs are depicted as ribbon structures. Side chains of pY694 and F706 are shown in stick representation. Magenta, carbon; red, oxygen; white, phosphorus. (**b**) Magnification of the hydrophobic interfaces in both monomers (cyan, M1; orange, M2). Residues of the hydrophobic interface and F706 (carbon magenta) are shown in stick representation. (**c**) Y694 phosphorylation of STAT5A-eYFP or STAT5A mutants stably expressed in HeLa T-REx HA-EpoR cells treated with sodium vanadate (1 mM, 1.5 h) as indicated. Cells were stimulated with 1 U/ml Epo for 30 min or left unstimulated. Whole cellular lysates (WCLs) were subjected to immunoblotting using antibodies against pY694/699-STAT5A/B and GFP. This blot and the following blots and gels were cropped to focus on relevant bands. The full-length blots and gels are provided with the Supplementary Information. (**d**) Native PAGE analysis of WCLs of HeLa T-REx HA-EpoR cells stably expressing STAT5A-eYFP or STAT5A mutants, treated with sodium vanadate (1 mM, 1.5 h) and/or Epo (1 U/ml, 30 min) as indicated. The native gel was analyzed by fluorescence scanning for the detection of YFP-tagged proteins. WCLs were subjected to immunoblotting using antibodies against pY694/699-STAT5A/B and GFP. (**e**) Quantification of native PAGE experiments. Normalized STAT5A dimerization levels and normalized pSTAT5/STAT5A ratios relative to stimulated cells expressing wild-type STAT5A were plotted. The data shown represent geometric means ± 95% confidence intervals of n = 4 independent experiments statistically evaluated by a paired ratio t-test with ***p < 0.005, **p < 0.01 and *p < 0.05. (**f**) Effect of sodium vanadate (V) treatment (1 mM, 1.5 h) on the nuclear accumulation of STAT5A-eYFP (yellow) and the indicated mutants in Epo-stimulated (1 U/ml, 30 min) HeLa T-REx HA-EpoR cells. Nuclei (blue) and the actin cytoskeleton (red) were stained with DRAQ5 and phalloidin, respectively. Unstimulated and Epo-stimulated cells served as controls (upper panel, left and middle). Scale bars: 20 μm.

In addition to the intermolecular interactions, our model revealed intramolecular contacts between the PTM and a hydrophobic interface in the SH2 domain that might play a fundamental role in the dimerization of STAT5A. In particular, F706 located in the very C-terminal part of the PTM establishes interactions with W631, F633, W641, L663, Y665 and L666 of its cognate SH2 domain (Fig. 3a,b and Supplementary Fig. 3). Specifically, the MD simulations revealed that F706^M1^ forms aromatic interactions mainly with F633^M1^ and W641^M1^, as shown by their large persistence of 55.1% and 60.0% during the simulation time (Supplementary Table 6) and transient ones with W631^M1^ (5.4% of persistence). In the opposite monomer, F706^M2^ forms aromatic interactions with F633^M2^, Y665^M2^ and W631^M2^ with occupancies of the hydrophobic contacts of 41.4%, 41.3% and 21.7%, respectively. Such a network of hydrophobic interactions substantially contributes to the recognition of F706 in their respective interfaces (Supplementary Table 6). The involved residues are not conserved across the STAT family indicating a candidate interface to target STAT5 specifically.

The relevance of the observed intramolecular hydrophobic interactions for STAT5A function was evaluated by assessing the Epo receptor (EpoR)/JAK2-mediated phosphorylation of Y694, dimer formation and nuclear accumulation of STAT5A being mutated at residue F706 (F706A) or in the hydrophobic acceptor interface (F633A, W641A and Y665A). These mutants were studied in comparison to STAT5A wild-type and the non-functional R618Q mutant. HeLa T-REx cells stably expressing eYFP-tagged STAT5A constructs and the EpoR revealed a severely impaired tyrosine phosphorylation of all mutants in response to Epo stimulation. Intriguingly, the inhibition of phosphatases by sodium vanadate treatment rescued the Epo-induced phosphorylation of STAT5A mutants F706A, F633A or Y665A demonstrating that the reduced phosphorylation of these mutants in the absence of vanadate is caused by phosphatase activity rather than dysfunctional receptor recruitment (Fig. 3c). Of note, vanadate treatment alone did not result in phosphorylation of any of the STAT5A constructs (Supplementary Fig. 4).

To study the impact of these mutations on STAT5A dimerization, blue-native PAGE was performed. Since phosphorylation at Y694 is a prerequisite for the formation of STAT5A dimers, lysates were analyzed after treatment of cells with Epo and vanadate. Phosphatase inhibition resulted in a 2.15-fold increase in dimerization of STAT5A wild-type in response to Epo stimulation, illustrating that the EpoR/JAK2/STAT5A signaling pathway in steady-state is highly counter-regulated by phosphatases. Surprisingly, while in response to vanadate treatment STAT5A proteins carrying the F706A mutation appeared to be phosphorylated markedly above reference levels (Epo stimulation of STAT5A wild-type in the absence of vanadate), their dimerization was severely impaired (−2.37-fold) (Fig. 3d,e). Likewise, the mutations F633A (−1.59-fold) and Y665A (−2.07-fold) located in hydrophobic interface of the SH2 domain, compromised dimerization, indicating that the intramolecular interaction between F706 and the hydrophobic interface is essential for dimerization of tyrosine-phosphorylated STAT5A and probably to maintain the phosphate by shielding it from phosphatases (Fig. 3d,e).

In contrast, vanadate treatment failed to restore the receptor-mediated phosphorylation of the STAT5A mutants F633A/Y665A, W641A and R618Q to reference levels (Fig. 3c–e), suggesting that these mutations severely affect the structural integrity of the SH2 domain and thus prevent appropriate recruitment to signaling-competent receptors.

In resting cells, STAT5 is evenly distributed between cytoplasm and nucleus. Formation of phosphorylated dimers is a requirement for nuclear accumulation of STAT5. Confocal imaging of fluorescently labeled STAT5A revealed that vanadate treatment resulted in a forced nuclear enrichment of the wild-type protein in erythropoietin-stimulated cells, while the investigated STAT5A mutants did not accumulate in the nucleus reflecting their impaired capability to form dimers (Fig. 3f). Taken together the data demonstrated that F706, an aromatic residue located in a region for which no structural information is available for any of the STAT proteins, is essential for the formation of activated STAT5A dimers. Moreover, tyrosine phosphorylated monomers that fail to dimerize undergo rapid dephosphorylation.

### Disease mutations N642H and T628S in the phosphotyrosine-SH2 domain interface improve phosphotyrosine-binding but require F706

**Figure 4:**
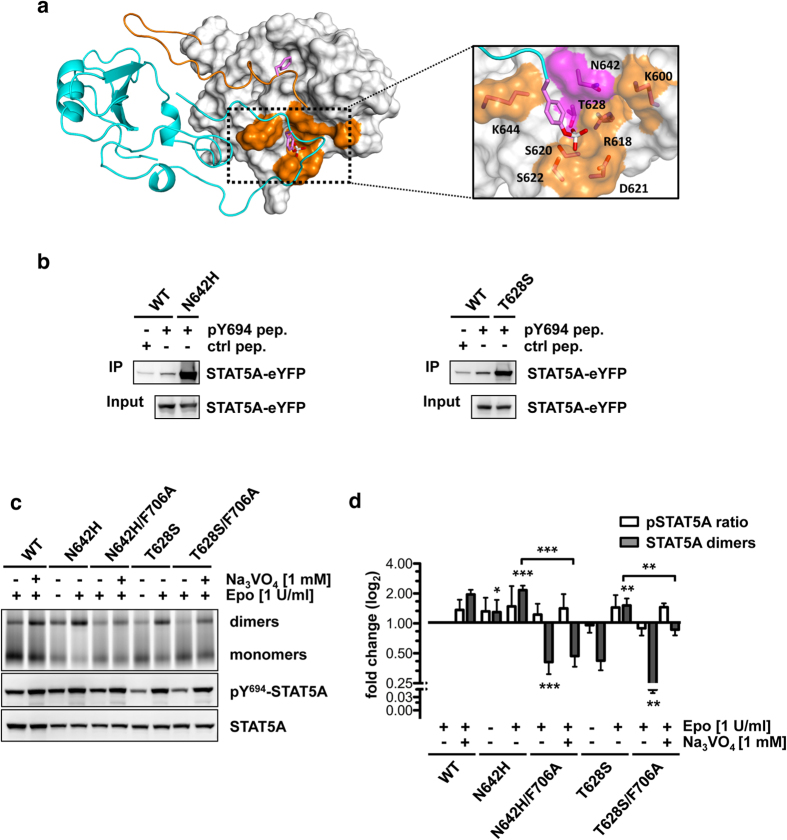
STAT5A mutations N642H and T628S cause constitutive activation and remain susceptible to F706 substitution. (**a**) STAT5A dimer interface. Monomer 1 is depicted as ribbon structure (cyan). The SH2 domain of monomer 2 is shown in surface representation (grey) with the CTS (res. 690–707) depicted as ribbon structure (orange). Side chains of pY694 and F706 are shown in stick representation. Magenta, carbon; red, oxygen; white, phosphorus. The phosphotyrosine-SH2 domain interface is magnified and residues contributing to phosphotyrosine-binding are shown in stick representation and orange surface color. Mutation-sites N642 and T628 are highlighted in magenta. (**b**) Peptide precipitation assay using WCLs of HeLa T-REx FRT cells expressing STAT5A-eYFP, STAT5AN642H-eYFP or STAT5AT628S-eYFP. Lysates were incubated with biotinylated control (F694) or phosphorylated (pY694) STAT5A peptides coupled to NeutrAvidin-Agarose. Precipitates and WCLs were analyzed by immunoblotting using a GFP-specific antibody. (**c**) Native PAGE analysis of WCLs of HeLa T-REx HA-EpoR cells stably expressing STAT5A-eYFP or STAT5A mutants, treated with sodium vanadate (1 mM, 1.5 h) and/or Epo (1 U/ml, 30 min) as indicated. The native gel was analyzed by fluorescence scanning for the detection of YFP-tagged proteins. WCLs were subjected to immunoblotting using antibodies against pY694/699-STAT5A/B and GFP. (**d**) Quantification of native PAGE experiments. Normalized STAT5A dimerization levels and normalized pSTAT5/STAT5A ratios relative to stimulated cells expressing wild-type STAT5A were plotted. The data shown represent geometric means ± 95% confidence intervals of n = 4 independent experiments statistically evaluated by a paired ratio t-test with ***p < 0.005, **p < 0.01 and *p < 0.05.

In a defined subset of aggressive lymphoid cancers, the somatic mutations N642H (βD5) and T628S (β-sheet C) have recently been identified, which map to the phosphotyrosine-binding pocket in interface 1 of the STAT5 SH2 domain (Figs 1e, and 4b). In particular, the N642H mutation was found at high frequencies in lymphomas of NK or γδ T-cell origin and in T-cell prolymphocytic leukemia (T-PLL) patients with high frequencies of 33.3% (7/21), 14.9% (14/94) and 22.0% (11/50), respectively^15^,^17^,^19^. Moreover, N642H mutations were detected at lower frequencies in T-cell acute lymphoblastic leukemia (T-ALL), pediatric T-ALL and fatal large granular lymphocytic leukemia (LGLL) (7.4% (5/68), 6.3% (19/301) and 1.9% (4/211), respectively)^14^,^16^,^18^. The T628S mutation was found at a frequency of 6.0% (3/50) in T-PLL patients^15^.

Peptide precipitation experiments using a peptide containing phosphorylated Y694 revealed that these mutations increase the binding affinity of the mutated SH2 domains to the phosphotyrosine motif (Fig. 4b). Blue-native PAGE analyses further confirmed the activating phenotype of STAT5A^N642H^ and STAT5A^T628S^ by the detection of dimers that appeared 2.10-fold and 1.48-fold increased in Epo-stimulated cells, suggesting that both mutations interfere with appropriate deactivation of the JAK-STAT signaling pathway (Fig. 4c,d). Moreover, STAT5A^N642H^ dimers appeared already considerably elevated in the absence of stimulation (1.27-fold) demonstrating that this mutation confers a stronger constitutive activation to STAT5 proteins compared to T628S (Fig. 4c,d).

Interestingly, the combination of activating N642H and T628S mutations with F706A resulted in significantly diminished STAT5A dimer levels in Epo-stimulated cells with fold changes of −2.47 and −4.51, respectively. Moreover, the dimerization of both double mutants appeared significantly impaired in vanadate treated cells compared to their single mutated counterparts (4.26-fold and 2.70-fold). Strikingly, phosphatase inhibition failed to restore STAT5A dimerization back to reference levels confirming that the phosphorylation of STAT5A is necessary yet not sufficient for dimerization (Fig. 4c,d). These findings identify interface 3 as a target region for the development of compounds against activating STAT5 mutations implicated in aggressive lymphoid cancers with poor prognosis.

### Mutations in the PTM-PTM interface enhance STAT5 dimerization

**Figure 5:**
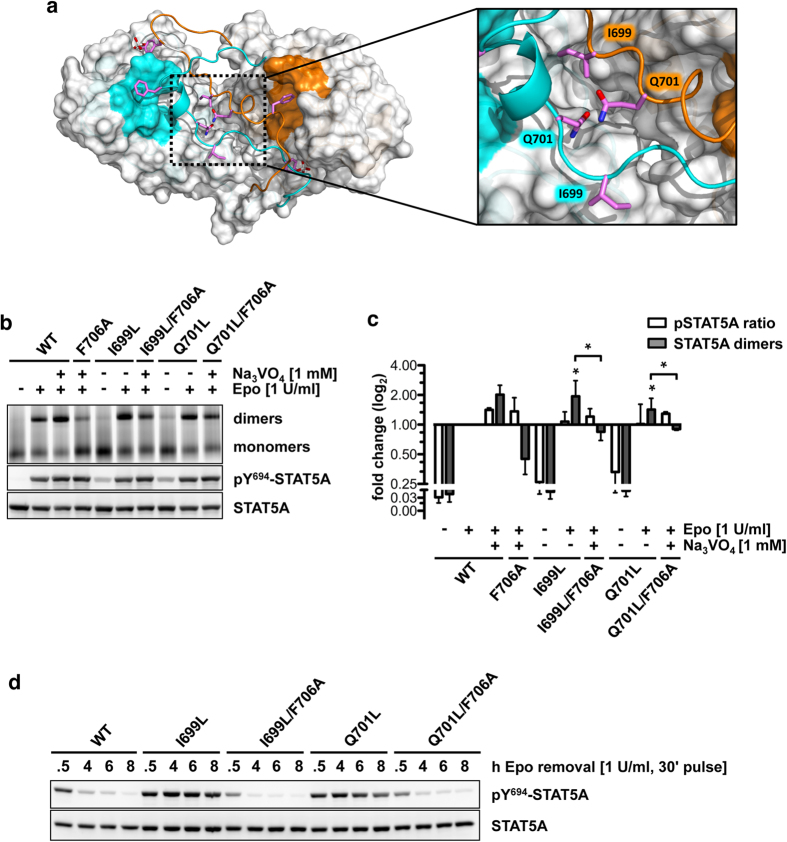
PTM mutations I699L and Q701L convey constitutive STAT5 activation through increased intermolecular PTM-PTM contacts. (**a**) STAT5A dimer interface (top view). The SH2 domains are depicted in surface representation (grey). The hydrophobic interfaces are highlighted in cyan (M1) and orange (M2), respectively. Both CTSs are depicted as ribbon structures. Side chains of pY694, I699, Q701 and F706 are shown in stick representation. Magenta, carbon; red, oxygen; white, phosphorus. The PTM-PTM interface is magnified and residues I699 and Q701 of both monomers are shown in stick representation. (**b**) Native PAGE analysis of WCLs of HeLa T-REx HA-EpoR cells stably expressing STAT5A-eYFP or STAT5A mutants, treated with sodium vanadate (1 mM, 1.5 h) and/or Epo (1 U/ml, 30 min) as indicated. The native gel was analyzed by fluorescence scanning for the detection of YFP-tagged proteins. WCLs were subjected to immunoblotting using antibodies against pY694/699-STAT5A/B and GFP. (**c**) Quantification of native PAGE experiments. Normalized STAT5A dimerization levels and normalized pSTAT5/STAT5A ratios relative to stimulated cells expressing wild-type STAT5A were plotted. The data shown represent geometric means ± 95% confidence intervals of n = 3 independent experiments statistically evaluated by a paired ratio t-test with ***p < 0.005, **p < 0.01 and *p < 0.05. (**d**) Tyrosine 694 phosphorylation kinetics of STAT5A-eYFP or the indicated STAT5A mutants stably expressed in HeLa T-REx HA-EpoR cells. Cells were pulse-stimulated with 1 U/ml Epo for 30 min and analyzed at the indicated time periods. WCLs were subjected to immunoblotting using antibodies against pY694/699-STAT5A/B and GFP.

Compared to the N642H mutation, the I699L mutation (I704L in STAT5B) appears at a lower frequency of 2.0% in lymphomas of NK or γδ T-cell origin (1/94)^17^ and T-ALL patients (1/68)^16^. Likewise, the Q701L mutation (Q706L in STAT5B) is present at a frequency of 2.0% (1/50)^15^ in T-PLL patients. I699 and Q701 map to the PTM at positions pY+5 and pY+7, respectively. Here, we analyzed the impact of these substitutions on STAT5A function in order to verify the relevance of intermolecular PTM-PTM contacts for dimerization (Fig. 5a). Blue-native PAGE analyses revealed an activating phenotype of both mutations in stimulated cells with an increase in dimerization of 1.96-fold and 1.43-fold compared to wild-type (Fig. 5b,c). In contrast to STAT5^N642H^, mutations in the PTM resulted only in a weak factor-independent constitutive activation, illustrating that both mutants are more sensitive to dephosphorylation similar to STAT5A^T628S^. The introduction of the F706A mutation into I699L and Q701L mutants again significantly diminished dimerization in stimulated cells compared to the corresponding single mutants (3.13-fold and 2.54-fold), despite boosted phosphorylation levels caused by vanadate treatment (Fig. 5b,c). Without phosphatase inhibition Y694 phosphorylation was markedly reduced (Supplementary Fig. 5). Notably, each of both double mutants partially rescued the dimerization defect of STAT5A^F706A^, suggesting that stabilizing intermolecular interactions in the PTM-PTM interface increase dimer stability, interfere with appropriate inactivation through STAT5-specific phosphatases and partially compensate for the loss of F706 (Fig. 5b). Nevertheless, phosphorylation kinetics revealed that the mutation of F706 to alanine effectively abolishes constitutive activation of STAT5A^I699L^ and STAT5A^Q701L^ in stimulated cells (Fig. 5d).

### Constitutive STAT5 activation by the Y665F mutation in the hydrophobic interface

**Figure 6:**
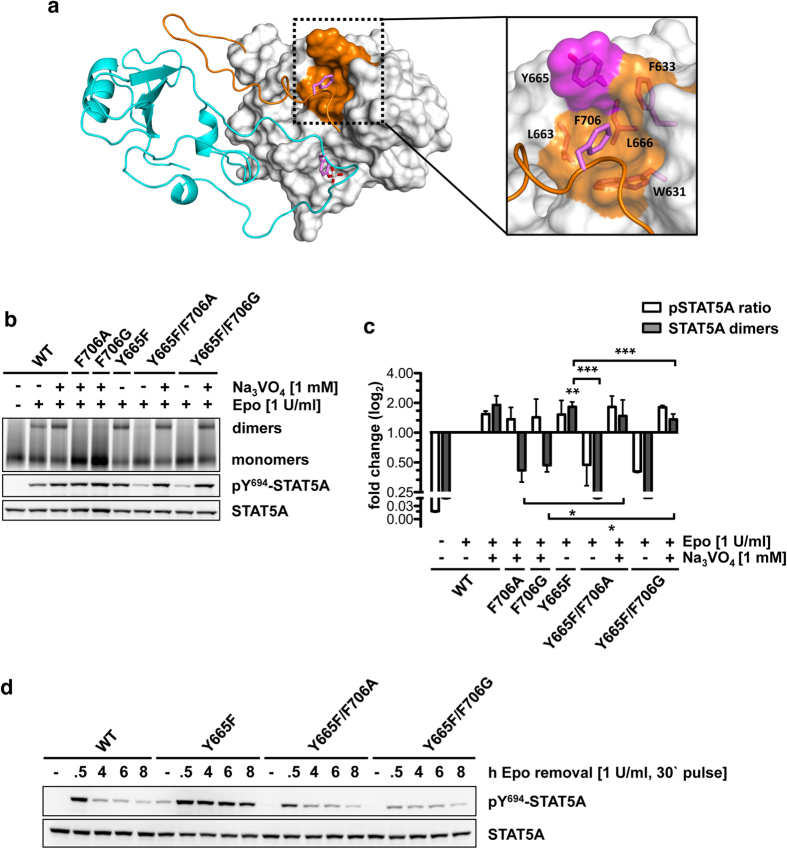
F706 is required for constitutive activity of the Y665F mutation. (**a**) STAT5A dimer interface (top view). The SH2 domains are depicted in ribbon (cyan, M1) or surface representation (grey, M2). The hydrophobic interface of M2 is highlighted in orange. Both CTSs are depicted as ribbon structures. Side chains of pY694, I699 and F706 are shown in stick representation. Magenta, carbon; red, oxygen; white, phosphorus. The F706 interface is magnified and residues F706, L663, Y665, F633, L666 and W631 are shown in stick representation. Mutation-site Y665 is highlighted in magenta. (**b**) Native PAGE analysis of WCLs of HeLa T-REx HA-EpoR cells stably expressing STAT5A-eYFP or STAT5A mutants, treated with sodium vanadate (1 mM, 1.5 h) and/or Epo (1 U/ml, 30 min) as indicated. The native gel was analyzed by fluorescence scanning for the detection of YFP-tagged proteins. WCLs were subjected to immunoblotting using antibodies against pY694/699-STAT5A/B and GFP. (**c**) Quantification of native PAGE experiments. Normalized STAT5A dimerization levels and normalized pSTAT5/STAT5A ratios relative to stimulated cells expressing wild-type STAT5A were plotted. The data shown represent geometric means ± 95% confidence intervals of n = 3 independent experiments statistically evaluated by a paired ratio t-test with ***p < 0.005, **p < 0.01 and *p < 0.05. (**d**) Tyrosine 694 phosphorylation kinetics of STAT5A-eYFP or the indicated STAT5A mutants stably expressed in HeLa T-REx HA-EpoR cells. Cells were pulse-stimulated with 1 U/ml Epo for 30 min and analyzed at the indicated time periods. WCLs were subjected to immunoblotting using antibodies against pY694/699-STAT5A/B and GFP.

The Y665F mutation has been identified in T-PLL, NK/γδ T-cell lymphomas, T-ALL and LGLL with frequencies of 6.0% (3/50)^15^, 2.1% (2/94)^17^, 1.5% (1/68)^16^ and 1% (2/211)^18^, respectively. Y665 is part of the hydrophobic acceptor interface for the intramolecular recognition of F706 in wild-type STAT5A (Fig. 6a). STAT5A^Y665F^ showed a significant 1.82-fold (p = 0.0020) increase in the amount of dimers in stimulated cells compared to reference levels, while vanadate treatment remained again insufficient to rescue pSTAT5A^F706A^ or pSTAT5A^F706G^ dimerization (Fig. 6b,c).

Similar to intermolecular disease mutations in the PTM-SH2 domain and PTM-PTM interfaces, upon combination of Y665F with either F706A or F706G dimers were hardly detectable with significant reductions of 5.94-fold (p = 0.0003) and 6.16-fold (p = 0.0007) in stimulated cells compared to the Y665F single mutant (Fig. 6b,c). Surprisingly, the inhibition of phosphatase activity by vanadate efficiently rescued the dimerization of STAT5A^Y665F^ carrying the F706A (3.90-fold) or F706G (3.50-fold) mutation, suggesting that the elimination of the Y665-hydroxyl group is sufficient to enable appropriate dimerization of STAT5A^F706A/G^. Nevertheless, these dimers remained highly susceptible for phosphatases. Therefore, it appears evident that besides its role in the dimerization process, binding of F706 to the hydrophobic interface is critical to protect STAT5A dimers from rapid inactivation (Fig. 6b,c). In accordance with this concept, phosphorylation kinetics of both double mutants confirmed a phenotype comparable to wild-type STAT5A (Fig. 6d).

## Discussion

The crystal structures of the activated STAT1 and STAT3 dimers were used to predict the structural determinants of the fully assembled SH2 domain interface of STAT5A by the combination of homology modeling and MD simulations. The resulting model, validated with experimental data, revealed novel and unique features of the dimerization interface of STAT5A and was used to unravel the impact of disease-related STAT5 mutations on protein structure and function.

Not unexpected, the model corroborates reciprocal phosphotyrosine-SH2 domain interactions as a conserved driver of STAT dimerization resulting in a grossly symmetric dimer^24^,^25^. However, by comparing the contribution of individual residues to the coordination of the phosphotyrosine residue in each of both interfaces, a local structural asymmetry was observed (Fig. 2). Likewise, MD simulations of STAT1 and STAT3 dimers, which were generated based on their corresponding crystal structures, identified large-scale motions of both dimers finally resulting in non-symmetric interfaces^27^. In general, local asymmetry in otherwise symmetric protein complexes is not unusual^28^,^29^. In STAT5A, such asymmetry might mirror the transient nature of the phosphotyrosine-SH2 domain interaction that has evolved for temporary release of the phosphotyrosine moiety to become accessible for dephosphorylation^30^.

The binding interface selectivity of SH2 domains is mediated by individual binding pockets, additional to the phosphotyrosine-binding site, that primarily recognize residues in the pY+1 and pY+3 positions located C-terminal to the phosphotyrosine residue. In most SH2 domains, the βD5 residue critically contributes to selectivity by recognition of the pY+1 position^31^,^32^. Surprisingly, in the dimer structures of STAT1 and STAT3, the βD5 residue (A630 and S636) coordinates the phenyl ring of phosphotyrosine by providing non-polar interactions rather than accepting the pY+1 residue^24^,^33^. An equal binding mode with N642 located in the βD5 position is preserved in the STAT5A simulation (Fig. 2b and Supplementary Table 3). Intriguingly, while electrostatic contacts established by threonine in the pY+3 position were conserved between STAT1 and STAT3, the presence of proline at the corresponding position in the PTM of STAT5A prevents a similar binding mode, indicating that the recognition of the pY+3 residue by STAT SH2 domains might serve as a first step to define binding interface selectivity.

In STAT1 and STAT3, the PTM forms a pair of cross-over connections with the opposite PTM significantly increasing the intermolecular contact area at the dimer interface^24^,^25^. These intermolecular PTM-PTM interactions were also present in the simulation of the STAT5A dimer interface (Fig. 2d). In STAT1 and STAT3, these interactions are predominantly mediated by residues located in loops present in the SH2 domain finally establishing a clamp-like architecture. The resulting tunnel-like arrangement provides structural guidance for the percolating PTM (Fig. 1f). The lack of these loops in STAT5A is compensated by a hydrophobic interface in the SH2 domain that functions as an intramolecular acceptor of F706 located in the PTM of STAT5A (Fig. 3a,b). Consequently, mutations of either donor (F706) or acceptor (F633, Y665) residues severely impaired the dimerization and subsequent nuclear accumulation of pSTAT5A, demonstrating that intramolecular interactions are indeed indispensable for appropriate dimerization.

In recent years, an increasing number of somatic STAT5 mutations has been identified in leukemia patients. The vast majority of lymphoma-associated STAT5 mutations such as N642H, T628S, I699L, Q701L and Y665F map to the SH2 domain and PTM of the transcription factor and are characterized by an activating phenotype^14^,^15^,^16^,^17^,^18^,^19^.

The mutations N642H and T628S reside in the phosphotyrosine-binding interface and peptide precipitations confirmed an increased binding affinity for both of the mutated SH2 domains suggesting that constitutive activity is a consequence of an impaired inactivation by STAT5 phosphatases (Fig. 4b,e). MD simulation data revealed that N642 contributes to the coordination of the phenyl ring of phosphotyrosine by providing non-polar contacts. Consequently, it is tempting to speculate that the substitution of the amide side chain of asparagine by an imidazole group stabilizes phosphotyrosine through a π-stacking mechanism^17^,^34^,^35^. Alternatively, hydrogen bond interactions formed between the NH-group of the imidazole side chain and phosphotyrosine might result in an increased affinity between the interaction partners (Fig. 2b, Supplementary Table 2). Likewise, T628 was identified to support phosphotyrosine recognition through the formation of hydrogen bond interactions (Fig. 2a, Supplementary Table 2). Conceivably, the substitution of the threonine methyl group by hydrogen in STAT5A^T628S^ directly strengthens phosphotyrosine coordination by the SH2 domain. Importantly, the improved phosphotyrosine-binding ability of both SH2 domains did not suffice to rescue dimerization of STAT5A proteins lacking F706, further strengthening the relevance of intramolecular interactions for STAT dimerization (Fig. 4c,d).

In addition to phosphotyrosine-SH2 domain interactions, intermolecular contacts between the two PTMs have been reported to be important mediators for the dimerization of STAT1 and STAT3^24^,^25^. In support of this concept, our data showed that the cancer-related activating mutations I699L and Q701L located in interface 2 of the STAT5A molecule indeed resulted in elevated dimerization levels, but remained highly susceptible to alteration of the F706 residue (Fig. 5c,d). Notably, STAT5A^I699L/F706A^ and STAT5A^Q701L/F706A^ exhibited a weaker dimerization defect compared to the F706A mutant providing evidence that L699 (pY+5) and L701 (pY+7) significantly support the dimerization of STAT5A. Hence, constitutive activation of STAT5A^I699L^ and STAT5A^Q701L^ is most likely a consequence of increased binding affinities between the two PTMs (Fig. 5c,d). Accordingly, mutation of I699 to alanine results in accelerated dephosphorylation of STAT5A (data not shown).

Finally, while intramolecular interactions of the PTM with the SH2 domain exist in STAT1 and STAT3^24^,^25^, their contribution to the dimerization process has not yet been addressed. Based on our simulation data, it appears conceivable that constitutive activity of STAT5A^Y665F^ is caused by increased intramolecular binding of F706. Blue-native PAGE analyses indeed demonstrated that STAT5A^F706A/G^ proteins carrying the Y665F mutation in the hydrophobic acceptor interface became highly susceptible to phosphatase activity and thus confirm the relevance of F706 in protecting STAT5A dimers from excessive deactivation (Fig. 6b,c).

Surprisingly, these experiments additionally revealed that F706A and F706G mutations only moderately compromised the dimerization potential of STAT5A^Y665F^, indicating that the Y665F mutation compensates the dimerization defect of STAT5A^F706A/G^. Possibly, F706 itself orchestrates the orientation of the aromatic components located in the hydrophobic pocket to allow for dimerization of STAT5A.

Conclusively, the data demonstrated that the presence of F706 is critical for the dimerization process of phosphorylated STAT5A. Additionally, the hydrophobic interactions protect the STAT5A dimer from rapid inactivation by phosphatases. In the light of the fact that the VHR phosphatase utilizes a phosphotyrosine motif to displace the PTM from the STAT5A SH2 domain, increased binding affinities in all three interfaces suffice to interfere with this particular mechanism and render STAT5 proteins constitutively active^36^. *Elumalai* and colleagues recently reported on the development of the STAT5 inhibitor Stafib-1 that might simultaneously engage the phosphotyrosine binding pocket and the hydrophobic interface^37^. Our study gives a rational explanation for the action of this inhibitor and strongly supports the notion that the pharmacological inhibition of F706-binding to the hydrophobic interface in combination with compounds occupying the phosphotyrosine-binding pocket in the SH2 domain, represents a promising strategy to develop potent and selective STAT5 inhibitors.

## Methods

### Homology modeling

The structural determinants of activated STAT5A dimer interface (residues 589–714, SH2 domain + CTS) were initially obtained by using homology modeling. The latter was based on the correspondent domains in the crystal structures of activated STAT1 dimer (PDB ID: 1BF5^25^), activated STAT3 dimer (PDB ID: 1BG1^24^), and inactive STAT5A monomer (PDB ID: 1Y1U^23^) (sequence identities 25.5%, 31.9%, and 100%, respectively). Additional templates were identified for the highly disordered nTAD domain by the BLAST webserver (http://blast.ncbi.nlm.nih.gov/Blast.cgi). These are bacteria CobB sirtuin (residues 260–266 of PDB ID: 1S5P^38^) and Parkia platycephala lectin 2 (residues 227–231 and 234–241 of PDB ID: 2GSJ^39^), with sequence identities of 26% and 37%, respectively. 200 models of the dimer were generated using the MODELLER 9v9 package^40^. They turned out to have 80% residues or more in the most favored regions of Ramachandran plots^41^, as shown by the Procheck program^42^. Specifically, the structures of activated STAT1 and STAT3 dimers were used only to retrieve the initial dimerization patterns of the STAT5A SH2 domain dimer.

### Molecular dynamics simulation

The homology model with the lowest DOPE (discrete optimized protein energy) scoring function^43^ as implemented in MODELLER was inserted into a water box with edge lengths of 64 Å, 73 Å and 84 Å. The protonation states of histidine residues (H604 of each monomer) were assigned to Nε nitrogen atom protonated according to the corresponding pKa values calculated by using the H^++^ webserver^44^. The system was neutralized by Na^+^ ions. Including water molecules, the system overall contained 39,901 atoms. It underwent MD simulations with AMBER ff99SB-ILDN force field^45^,^46^,^47^,^48^ using the GROMACS 4.6.5 code^49^. The Åqvist potential^50^ and TIP3P model^51^ were used for the ions and for the water molecules, respectively. All bond lengths were constrained by LINCS algorithm^52^. Periodic boundary conditions were applied. Electrostatic interactions were calculated using the Particle Mesh-Ewald (PME) method^53^, and van der Waals and Coulomb interactions were truncated at 1.0 nm. The system underwent 1,000 steps of steepest-descent energy minimization with 1,000 kJ·mol−1 Å−2 harmonic position restraints on the protein, followed by 2,500 steps of steepest-descent and 2,500 steps of conjugate-gradient minimization without restraints. The system was then gradually heated from 0 K up to 298 K in 20 steps of 2 ns. After that, 2000 ns long productive MD simulations were carried out in the NPT ensemble. The most representative structure was identified by the cluster analysis^49^ over the equilibrated trajectories, ranging from 1500 ns to 2000 ns. To assess the convergence of the simulated trajectory we considered their projections on the top essential dynamical spaces obtained from a standard covariance analysis. Following Hess’s criterion^26^, these projections were next compared with those expected for a random reference. The observed negligible overlap (i.e. cosine content close to 0, see Supplementary Table 1) confirms a posteriori adequate sampling of STAT5A conformations around the equilibrium position in the last 500 ns. Hydrogen bond interactions were defined to be present if the atomic distance between the acceptor and donor atoms is below 0.35 nm and the angle among the hydrogen-donor-acceptor atoms are below 30 degree. Hydrophobic interactions were defined to be present if the center-of-mass distance between side chains are smaller than 0.5 nm^54^.

### Hormones and antibodies

Erythropoietin (Roche, Basel, Switzerland) was used at a concentration of 1 U/ml. Anti-pY694-STAT5 (#9351, Cell Signaling, Berverly, USA) and anti-GFP (600-101-215, Rockland, Gilbertsville, USA) were used for immunoblotting. Anti-rabbit and anti-goat antibodies conjugated to horseradish peroxidase (HRP) were ordered from, DAKO (Glostrup, Denmark).

### Plasmid constructs

The cDNA of murine STAT5A was either cloned into pLentiLox (Vector Core, University of Michigan, USA) or pcDNA3.1 (Invitrogen, Paisley, UK) expression vectors and fused to the cDNA of the enhanced yellow fluorescent protein (eYFP). The cDNA of the human erythropoietin receptor (EpoR) was cloned into the pcDNA5/FRT/TO expression vector and fused to the cDNA of the hemagglutinin tag (HA). Fluorescent proteins were fused to the C-terminus of the target proteins, whereas the HA-tag was fused to the N-terminus of the human EpoR. Point mutants of STAT5A were generated with PCR based QuikChange^®^ XL Site-Directed Mutagenesis Kit according to manufacturer’s instructions (Agilent Technologies, Santa Clara, CA, USA).

### Cell culture, transfection and inducible cell lines

HeLa T-Rex (Invitrogen, Paisley, UK) cells were grown in Dulbecco’s modified Eagle’s medium (DMEM)/GlutaMAX (Gibco, Paisley, UK). Transient transfection of HeLa T-REx cells was performed using Trans IT^®^-LT1 (Mirus, Madison, USA) according to manufacturer’s instructions. Stable transfection of HeLa T-Rex cells for the inducible expression of HA-EpoR was performed with the Flp-In system (Invitrogen, Paisley, UK) using 250 μg/ml hygromycin B (PAA, Austria) and 15 μg/ml blasticidin (Invitrogen, Toulouse, France) for selection. The expression of HA-EpoR was induced with 5 ng/ml doxycycline (Sigma, St. Louis, USA) for 24 h. All media were supplemented with 10% FCS (Life Technologies, Darmstadt, Germany) and 25 U/ml penicillin/streptomycin (Lonza, Verviers, Belgium). Cells were incubated at 37 °C in a water-saturated atmosphere with 5% CO_2_. Phosphatase inhibition was carried out by sodium vanadate (Na_3_VO_4_) treatment (1 mM) for 2 h.

### Lentiviral mediated stable transduction

To achieve stable and constitutive expression of STAT5A constructs in inducible HeLa T-Rex cells, self-inactivating lentiviral constructs were used according to the guidelines of the RNAi consortium. Briefly, the cDNA of STAT5A-eYFP and the point mutants of STAT5A-eYFP were cloned into the lentiviral pLentiLox expression vector downstream of the CMV promotor (Vector Core, University of Michigan, USA). HEK293-T packing cells were seeded on 6-cm dishes on to be 80–90% confluent at the time of transfection. On the second day, HEK293-T cells were transiently co-transfected with the lentiviral vector, psPAX2 (packing plasmid) and pMD2.G (envelope plasmid). Eighteen hours after transfection the culture medium was replaced by high serum growth medium containing 30% FCS. Viruses were harvested 24 hours after medium exchange. For the infection of HeLa T-Rex cells, virus-containing supernatant was supplemented with 8 μg/ml polybrene (Sigma, St-Louis, USA) prior to infection.

### Preparation of cell lysates, SDS-PAGE and immunoblotting

HeLa T-REx cells were washed with PBS (137 mM NaCl, 2.5 mM KCl, 8 mM Na_2_HPO_4_, 1.5 mM KH_2_PO_4_, adjusted to pH 7.4) once and lysed with RIPA lysis buffer (50 mM Tris–HCl, pH 7.4, 150 mM NaCl, 1 mM EDTA, 0.5% Nonidet P-40, 1 mM NaF, 15% glycerol) supplemented with phosphatase/protease inhibitors (1 mM Na_3_VO_4_, 0.5 mM EDTA, 0.25 mM phenylmethylsulfonylfluoride (PMSF), 5 μg/ml aprotinin, 2.5 μg/ml leupeptin) according to standard procedures. The proteins were separated by SDS-PAGE and transferred to a PVDF membrane with subsequent immunodetection using specific antibodies. Primary and HRP-conjugated secondary antibodies were diluted in TBS-N buffer (20 mM Tris–HCl, pH 7.5, 135 mM NaCl, 0.1% Nonidet P-40). Membrane-bound antibody complexes were detected by chemiluminescence (ECL, Millipore, Billerica, MA, USA).

### Blue Native PAGE and detection of enhanced YFP

Dimerization of fluorescently eYFP-labeled STAT5A constructs was analyzed in HeLa T-REX HA-EpoR cells. Cell lysis was performed under native conditions using the lysis buffer of the NativePAGETM Sample Prep kit (Invitrogen Paisley, UK) supplemented with 2% digitonin (Sigam, St.Louis, USA). The lysates were cleared by centrifugation and incubated with Coomassie brilliant blue G-250 and separated over night at 4 °C using NativePAGETM Bis-Tris gel system with a gradient polyacrylamide gel (4–16%) according to the manufacturer’s instructions. Dimerization of STAT5A-eYFP constructs was analyzed by detection of the eYFP fluorescence with a typhoon fluorescence scanner (GE Healthcare, Germany) by excitation with a 488 nm laser line. The emission was detected using a 515–555 nm bandpass filter.

### Peptide precipitation assay

Transiently transfected HeLa T-REx parental cells were lysed with storage buffer (150 mM NaCl, 50 mM Tris–HCl, pH 7.5, 0.1 mM EDTA, 10% glycerol, 0.5% Nonidet P-40) supplemented with phosphatase/protease inhibitors. Lysates were cleared by ultracentrifugation (100.000 g, 30′, 4 °C). Peptides corresponding to the phosphotyrosine motif of STAT5 (b-tat-AKAVDGpYVKPQIKQVVPEFVNASADAG-a) were synthesized with >95% purity, modified by N-terminal biotinylation (b-) and C-terminal amidation (-a) and contain the HIV tat protein derived GRKKRRQRRRPQ (tat) sequence at the N-terminus. 25 nmol of the peptides were coupled to NeutrAvidin Agarose Resin (Thermo Scientific, Pittsburgh, PA, USA) for 2 h at 4 °C, washed twice with storage buffer and incubated with 1 mg of the lysate over night at 4 °C. Peptide/protein complexes were washed 3 times with storage buffer for 10 minutes and the precipitates were analyzed by SDS-PAGE and immunoblotting.

### Fluorescence microscopy

HeLa T-REx cells expressing fluorescently labeled fusion proteins were grown on glass cover slips. After paraformaldehyde (3.7%) fixation cells were stained with DRAQ5 (Biostatus, UK) and Phalloidin-Atto 550 (Sigma, St. Louis, USA) to label nuclei and actin, respectively. Cover slips were mounted with ImmuMount (Thermo Scientific, Pittsburgh, PA) as has been described previously^55^. Fluorescence images of adherent cells were generated with a Zeiss LSM 710 confocal microscope (Zeiss, Jena, Germany), using the Zeiss LD C-apochromat 40x/1.1 water objective. Images represent confocal slices of approximately 1 μm and were analyzed with the ZEN 2012 software (Zeiss, Jena, Germany).

Information on statistics and sample numbers is given in the corresponding figure legends.

## Additional Information

**How to cite this article**: Fahrenkamp, D. *et al*. Intramolecular hydrophobic interactions are critical mediators of STAT5 dimerization. *Sci. Rep.* **6**, 35454; doi: 10.1038/srep35454 (2016).

## Supplementary Material

Supplementary Information
