# Supplementary material for: Intramolecular hydrophobic interactions are critical mediators of STAT5 dimerization
Source: Sci Rep. 2016 Oct 18;6:35454. doi: 10.1038/srep35454 (PMC5067585; doi:10.1038/srep35454)

Supplementary Information

Intramolecular hydrophobic interactions are critical mediators of STAT5 dimerization

Dirk Fahrenkamp, Jinyu Li, Sabrina Ernst, Hildegard Schmitz-Van de Leur, Nicolas Chatain, Andrea Küster, Steffen Koschmieder, Bernhard Lüscher, Giulia Rossetti, Gerhard Müller-Newen

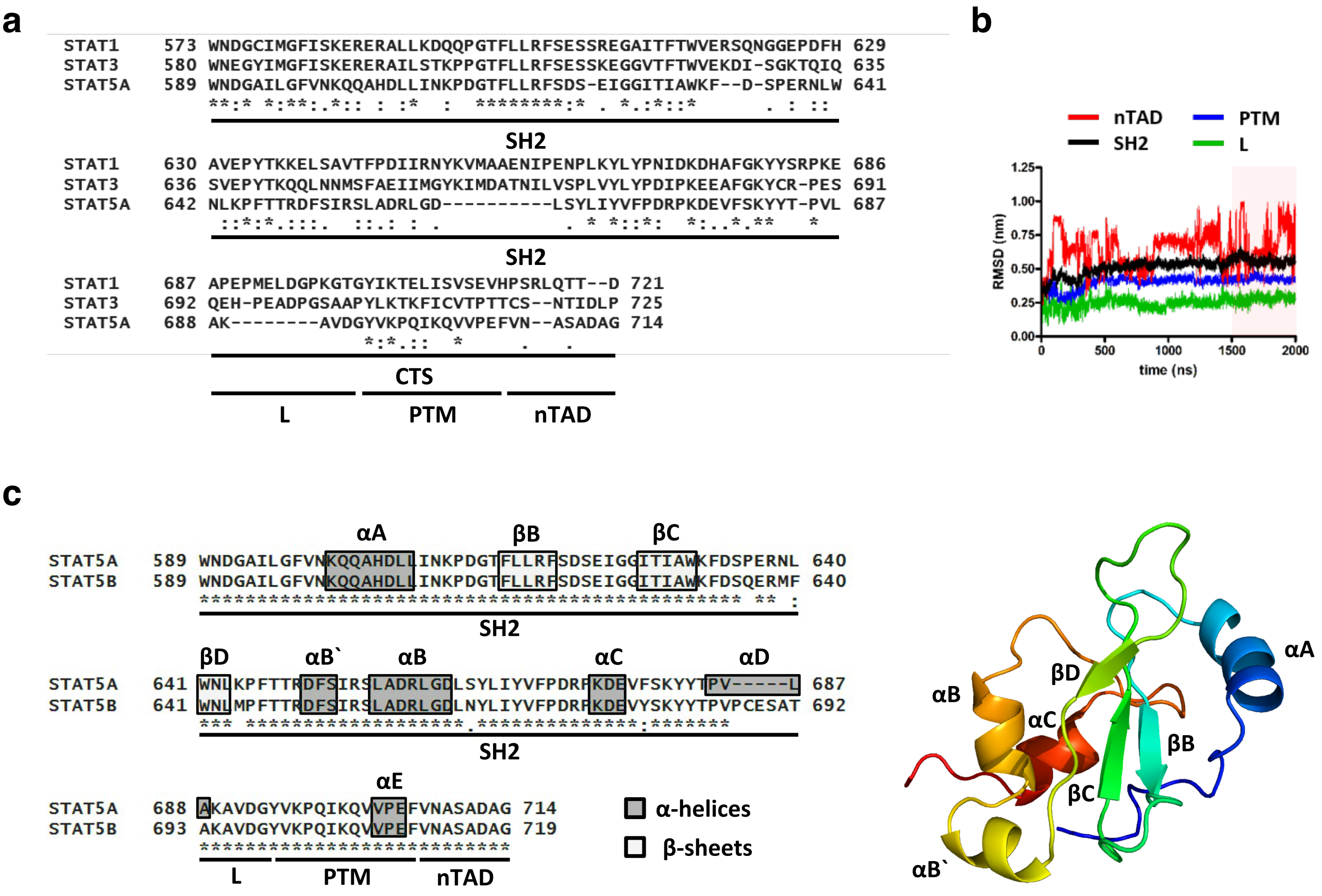

Supplementary Figure 1

Sequence similarities of STAT SH2 domains and structural features of the STAT5A SH2 domain. (a) Sequence similarity between the SH2 domains of human STAT1, STAT3 and STAT5A. Multiple sequence alignment of the indicated SH2 domains and the C-terminal tail segment (CTS) including the linker (L), phosphotyrosine motif (PTM) and N-terminal part of the TA domain (nTAD), was performed using T-Coffee[6]. (*) conserved residues (:) strong similarity (.) weak similarity.

(b) Backbone atoms` RMSD (in nm) relative to the initial structure of the activated STAT5A dimer interface for the SH2 domains (black line), L (green line), PTM (blue line) and nTAD (red line) of M1 and M2 as a function of simulation time. Red area: Time windows of the MD simulation used to generate the most representative model by cluster analysis.

(c) Left: Sequence alignment and secondary structure elements of the human STAT5A (PDB ID: 1y1u) and STAT5B SH2 domains. (*) conserved residues, (:) strong similarity, (.) weak similarity. Right: Rainbow representation of the ribbon structure of the modelled STAT5A SH2 domain (representative model). The core of the activated STAT5A SH2 domain (residues: 589-687) features a central anti-parallel β-sheet (βB, βC and βD) flanked by adjacent α-helices (αA, αB`, αB, αC and αD). With the exception of the presence of α-helix αD, the above highlighted secondary structures are preserved in the crystalized SH2 domain of inactive STAT5A.

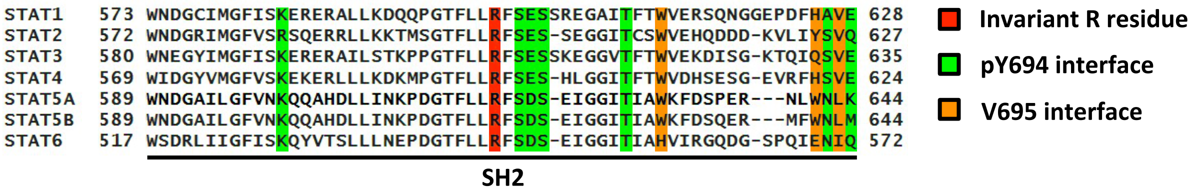

Supplementary Figure 2

Conserved residues contributing to intermolecular PTM/SH2 domain interactions in STAT dimer interfaces according to STAT1, STAT3 and STAT5A (Becker 1998, Chen 1998). Multiple sequence alignment of STAT SH2 domains was performed using ClustalW2[30]. The invariant and highly conserved arginine residue critical for the recognition of the phosphate group of phosphotyrosine and residues that support the binding of phosphotyrosine are highlighted in red and green background colors, respectively. Residues involved in the binding of V695 (pY+1) in the SH2 domain of STAT5A are depicted in orange.

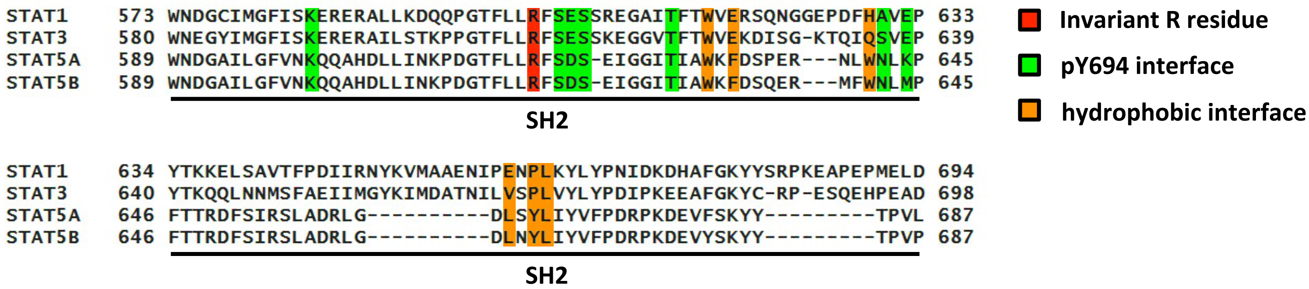

Supplementary Figure 3

Hydrophobic residues that contribute to intramolecular F706-binding in the STAT5A SH2 domain. Multiple sequence alignment of the STAT SH2 domains was performed using ClustalW2[30]. The invariant and highly conserved arginine residue critical for the recognition of the phosphate group of phosphotyrosine and residues that support the binding of phosphotyrosine are highlighted in red and green background colors, respectively. Residues involved in the intramolecular binding of F706 in the SH2 domains of STAT5A and STAT5B are depicted in orange.

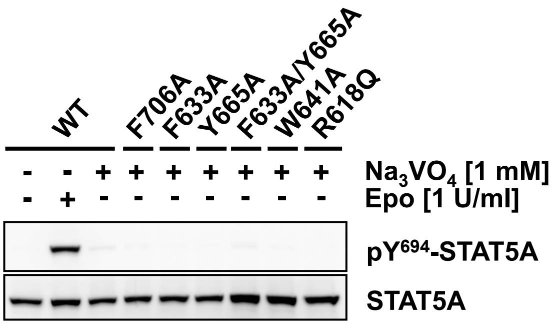

Supplementary Figure 4

STAT5 Y694 phosphorylation is hardly detectable in sodium vanadate treated cells. HeLa T-Rex EpoR cells expressing the indicated STAT5A mutants were treated with 1 mM sodium vanadate for 1.5 h. WCLs were analyzed for the phosphorylation status of STAT5A Y694. Unstimulated and Epo-stimulated cells (1 U/ml, 30 min) served as controls.

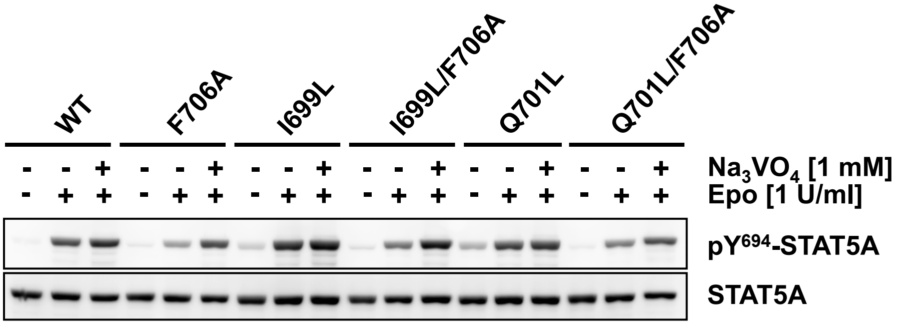

Supplementary Figure 5

The F706A mutation attenuates the phosphorylation of Y694 in STAT5A^{I699L} and STAT5A^{Q701L}. HeLa T-REx HA-EpoR cells stably expressing the indicated STAT5A-eYFP fusion proteins were treated with 1 mM sodium vanadate for 1.5 h or left untreated. Stimulation was carried out using 1 U/ml Epo for 30 min as indicated. Untreated cells served as controls. WCLs were analyzed for the phosphorylation status of STAT5A Y694.

Supplementary Table 1.

Hess's cosine content analysis of three principal components (PC1-PC3) obtained from mass weighted covariance analysis[4].

ID	Cosine content
PC1	0.147
PC2	0.126
PC3	0.175

Supplementary Table 2.

Residue interactions in the phosphotyrosine-SH2 domain interfaces of STAT5A

ID	Res. ^{M1}	Atom ^{M1}	Res. ^{M2}	Atom ^{M2}	Distance (nm) ^{a}	Distance (nm) ^{b}	Occupancy (%)^{c}
-	pY694		K600			0.86 ± 0.27	10.0
h1	pY694	O1P	R618	NH1	0.33 *	0.32 ± 0.01	100
h2	pY694	O1P	R618	NH2	0.28 *		
h3	pY694	O3P	R618	NH1	0.29		
h4	pY694	O3P	R618	NH2	0.35		
h5	pY694	O3P	S620	OG	0.33 *	0.35 ± 0.04	89.2
h6	pY694	OG	S622	OG	0.30 *	0.46 ± 0.15	66.5
h7	pY694	O2P	S622	OG	0.26 *		
h8	pY694	O3P	T628	OG1	0.25 *	0.42 ± 0.02	10.3
n1	pY694	CD2	N642	CB	0.35	0.61 ± 0.03	100
n2	pY694	CD1	K644	CB	0.50	1.17 ± 0.06	39.8
h9	K600	NZ	pY694	O1P	0.27 *	0.47 ± 0.14	55.6
h9	K600	NZ	pY694	O3P	0.35		
h10	R618	NH2	pY694	OG	0.28 *	0.37 ± 0.01	97.0
h11	R618	NH1	pY694	O3P	0.28 *		
h12	R618	NH2	pY694	O3P	0.29 *		
h13	S620	OG	pY694	O2P	0.25 *	0.48 ± 0.12	42.7
h14	S620	OG	pY694	O3P	0.34		
-	S622		pY694			0.67 ± 0.16	0.42
-	T628		pY694			0.53 ± 0.03	0.10
n3	N642	CB	pY694	CD1	0.50	0.73 ± 0.08	84.6
n4	K644	CD	pY694	CD2	0.35	0.41 ± 0.03	100

h: hydrogen bond

n: non-polar interaction

Res: Residue

M1/M2: Residues or atoms belong to monomer 1 / monomer 2

*: Hydrogen bonds illustrated in Fig. 2A.

a: Atom distances (h1-h14), calculated from the most representative model of the MD simulation

b: COM distances ± standard deviation (SD), calculated on the equilibrium trajectory of the MD simulation

c: Occupancy of the formation of hydrogen bonds across the equilibrium trajectory

Supplementary Table 3.

SH2 domain residues coordinating phosphotyrosine in STAT1, STAT3 and STAT5A

Pos.	STAT5A	STAT1	STAT3
αA2	K600	K584	K591
βB5	R618	R602	R609
βB7	S620	S604	S611
BC1	D621	-	E612
BC2	S622	S606	S613
βC1 *	T628	T613	S620
βD5 *	N642	A630	S636
βD7 *	K644	E632	E638

Modified from Gianti & Zauhar 2015 J Comput Aided Mol Des 29, 451-70

Residue annotation according to Songyan et al. 1993 Cell 72, 767-78; Sheinerman et al. 2003, J Mol Biol 334, 823-41

Supplementary Table 4.

Hydrogen bonds in the PTM-PTM interface of STAT5A

ID	Res. M1	Atom M1	Res. M2	Atom M2	Distance (nm) a	Occupancy (%) b
h1	Q698	N	Q701	O	0.29 ± 0.01	99.9
h2	Q698	O	Q701	NE2	0.29 ± 0.01	99.9
h3	K700	O	Q701	NE2	0.29 ± 0.02	99.0
h4	E705	OE1	I699	N	0.34 ± 0.05	62.7
h5	E705	OE2	Q698	N	0.33 ± 0.06	72.9

h: Hydrogen bond interactions, Res: Residue

a: Atom distances (h1-h5) + SD, calculated on the equilibrium trajectory of the MD simulation

b: Occupancy of the formation of hydrogen bonds (donor-acceptor distance below 0.35 nm and angle among the hydrogen-donor-acceptor atoms below 30 degree) across the equilibrium trajectory

Supplementary Table 5.

Hydrophobic interactions in the PTM-PTM interface of STAT5A

ID	Res. M1	Res. M2	Distance (nm) a	Occupancy (%) b
h1	P697	V702	0.42 ± 0.08	78.3
h2	V702	I699	0.44 ± 0.07	53.2

h: Hydrophobic interactions, Res: Residue

a: COM distances (h1 and h2) + SD, calculated on the equilibrium trajectory of the MD simulation

b: Occupancy of the formation of hydrophobic interactions (COM distance below 0.45 nm) across the equilibrium trajectory

Supplementary Table 6.

Interactions of F706 with residues in the hydrophobic interface of the STAT5A SH2 domain

ID	M	Res. [1]	Res. [2]	Distance (nm) [a]	Occupancy (%) [b]	Angle (θ) [c]
h1	1	F706	W631	0.72 ± 0.15	5.4	56.8 ± 11.0
h2	1	F706	F633	0.52 ± 0.13	55.1	18.8 ± 7.0
h3	1	F706	W641	0.48 ± 0.07	60.0	26.1 ± 11.6
-	1	F706	Y665	1.51 ± 0.15	0.0	-
h4	2	F706	W631	0.58 ± 0.12	21.7	28.6 ± 10.6
h5	2	F706	F633	0.68 ± 0.16	41.4	30.6 ± 15.5
-	2	F706	W641	1.04 ± 0.12	0.0	-
h6	2	F706	Y665	0.57 ± 0.12	41.3	50.4 ± 22.2

h: Hydrophobic interactions, Res: Residue

a: COM distances + SD, calculated on the equilibrium trajectory of the MD simulation

b: Occupancy of the formation of non-polar interactions across the equilibrium trajectory

c: Dihedral angle (θ) between stacking planar side chains. The planar side chains form parallel displaced stacking when θ = 0°; T-shaped stacking when θ = 90°; parallel staggered stacking when 0° < θ < 90°.

Supplementary Information

Full-length Western blots and gels

Figure 3c, upper panel

Figure 3c, lower panel

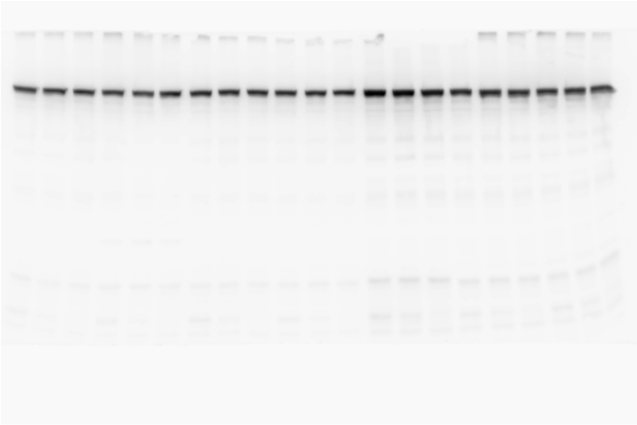

Figure 3d, upper panel (fluorescence scan of native PAGE to detect STAT5A-YFP)

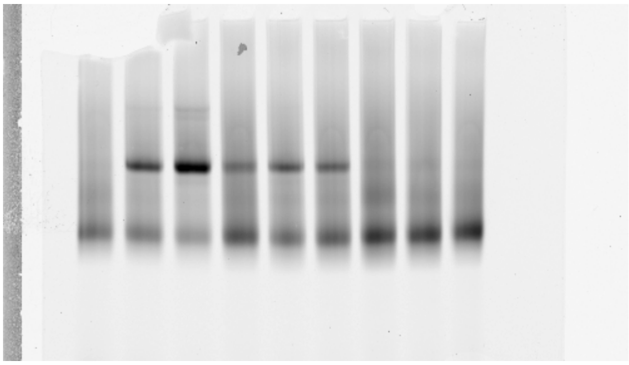

Figure 3d, middle panel

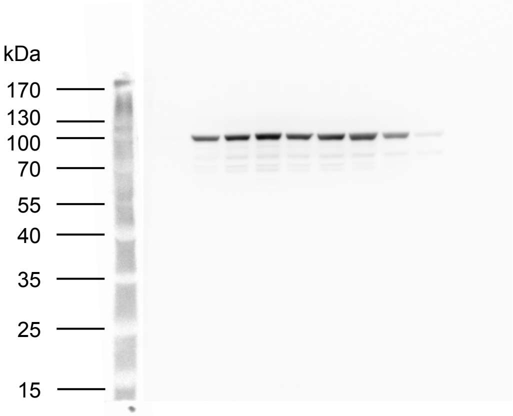

Figure 3d, lower panel

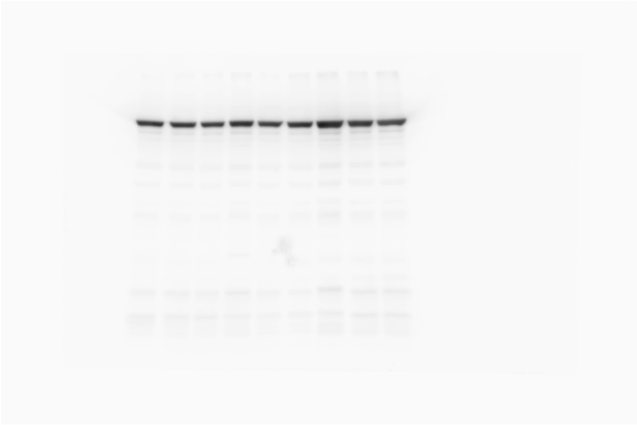

Figure 4b, left panel (N642H)

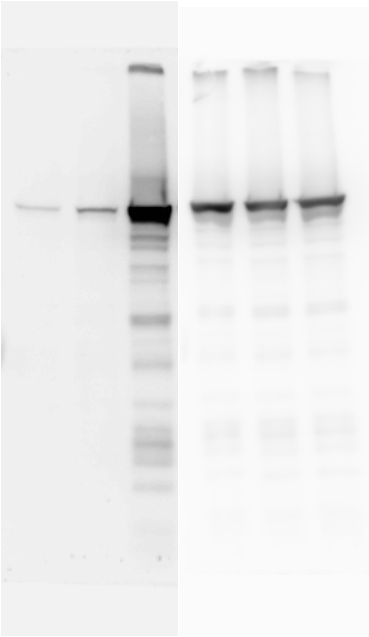

Figure 4b, right panel (T628S)

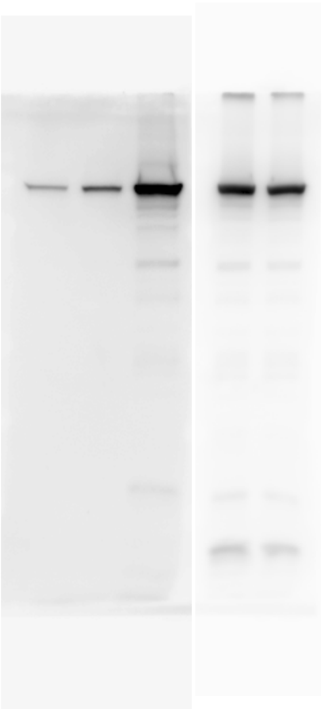

Figure 4c, upper panel (fluorescence scan of native PAGE to detect STAT5A-YFP)

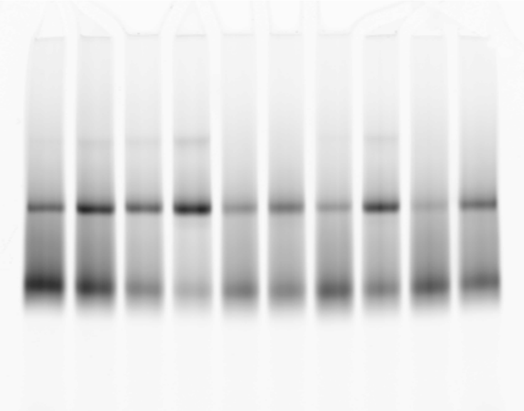

Figure 4c, middle panel

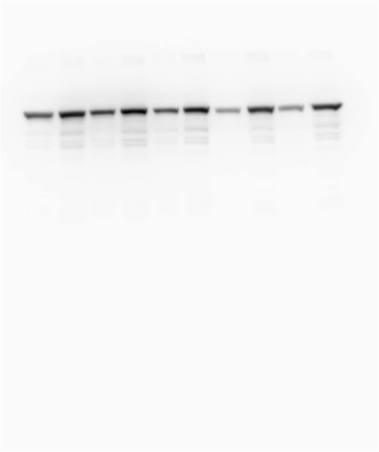

Figure 4c, lower panel

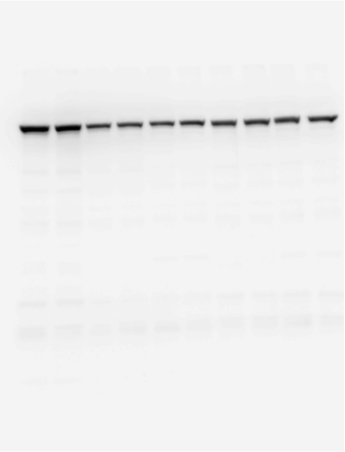

Figure 5b, upper panel (fluorescence scan of native PAGE to detect STAT5A-YFP)

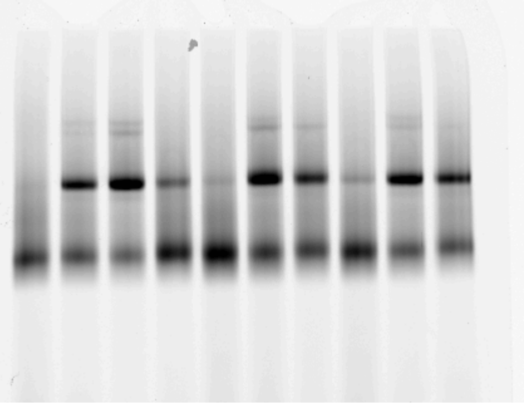

Figure 5b, middle panel

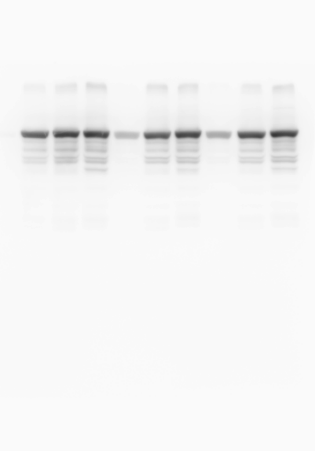

Figure 5b, lower panel

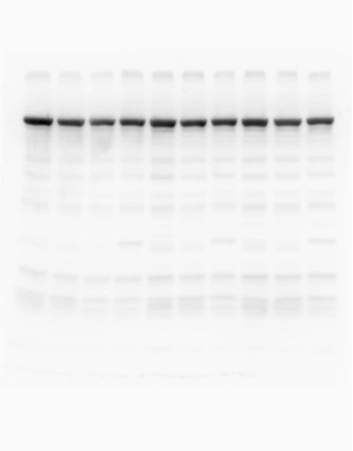

Figure 5d, upper panel

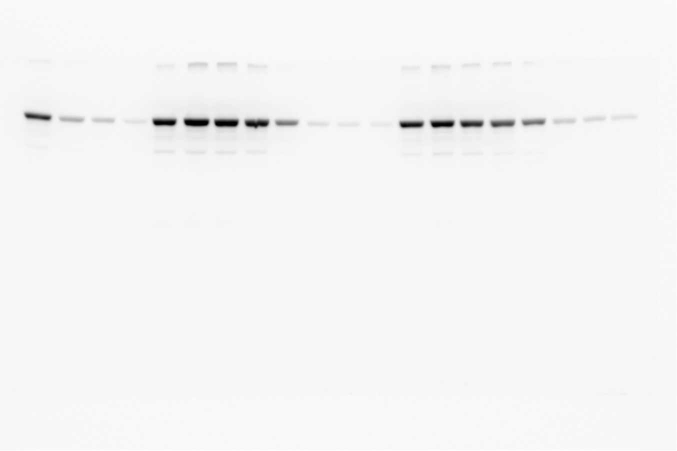

Figure 5d, lower panel

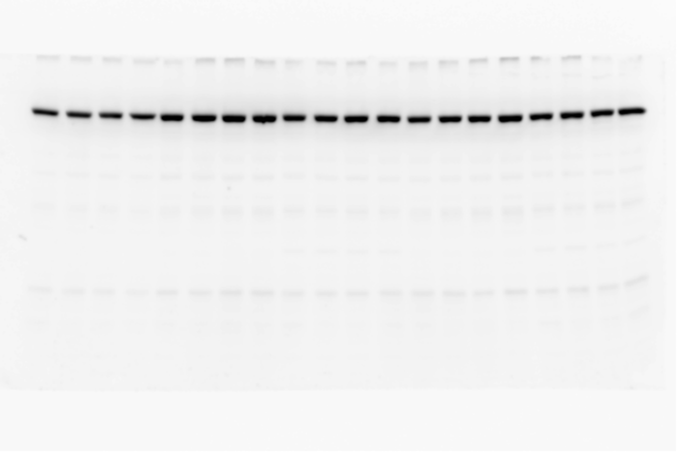

Figure 6b, upper panel (fluroescence scan of native PAGE to detect STAT5A-YFP)

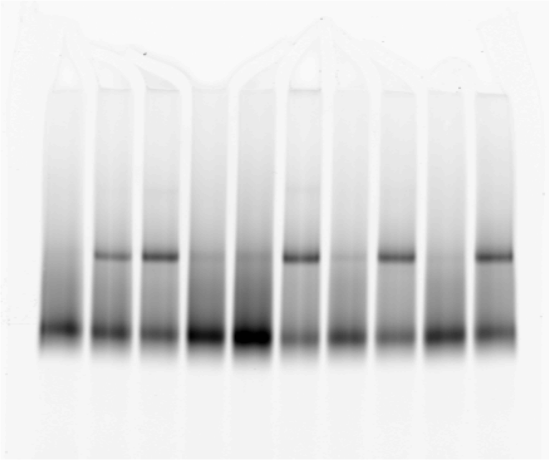

Figure 6b, middle panel

Figure 6b, lower panel

Figure 6d, upper panel

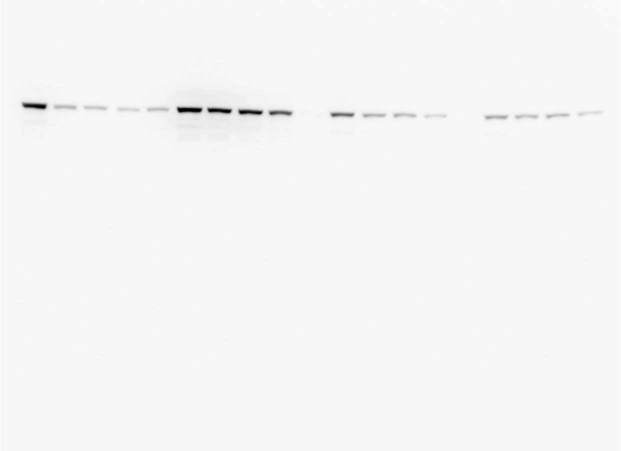

Figure 6d, lower panel

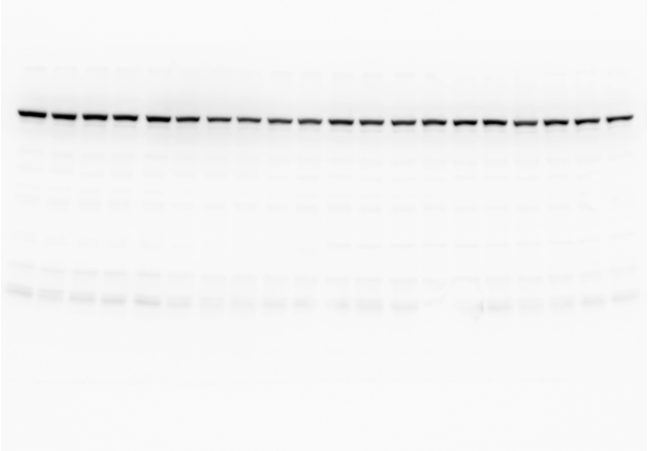

Suppl. figure 4, upper panel

Suppl. figure 5, upper panel

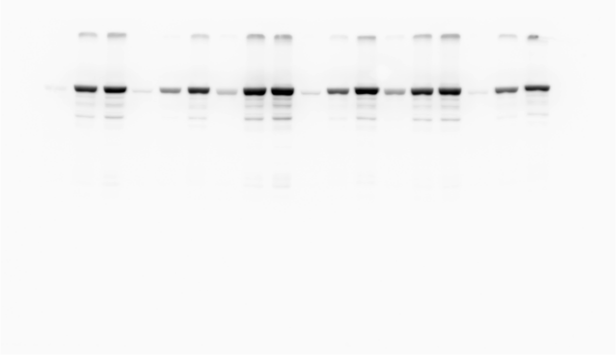

Suppl. figure 4, lower panel

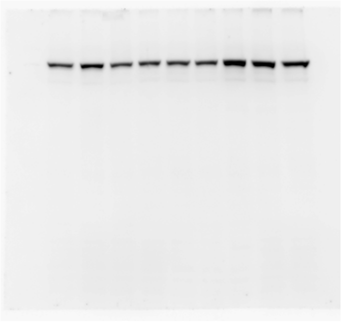

Suppl. figure 5, lower panel

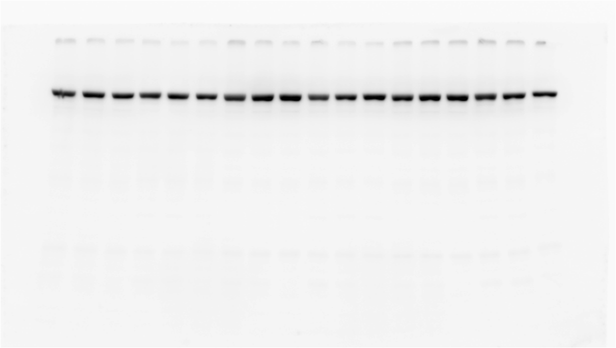

Supplement: Supplementary Information [file srep35454-s1.pdf]
